# Cross-Sectional Study of the Prevalence and Symptoms of Urinary Incontinence among Japanese Older Adults: Associations with Physical Activity, Health-Related Quality of Life, and Well-Being

**DOI:** 10.3390/ijerph18020360

**Published:** 2021-01-06

**Authors:** Noriaki Maeda, Yukio Urabe, Yuta Suzuki, Daigo Hirado, Masanori Morikawa, Makoto Komiya, Rami Mizuta, Koichi Naito, Taizan Shirakawa

**Affiliations:** 1 Department of Sports Rehabilitation, Graduate School of Biomedical and Health Sciences, Hiroshima University, Hiroshima 734-8553, Japan; yurabe@hiroshima-u.ac.jp (Y.U.); yt.suzuki28@gmail.com (Y.S.); m-masanori@hiroshima-u.ac.jp (M.M.); makoto-komiya@hiroshima-u.ac.jp (M.K.); dorami7572@gmail.com (R.M.); 2 Department of Rehabilitation, Matterhorn Rehabilitation Hospital, Hiroshima 737-0046, Japan; daigo0417tennis@gmail.com; 3 Department of Physical Therapy, Hakuho College, Nara 636-0011, Japan; kouichi2007creha@yahoo.co.jp; 4 Department of Orthopedics, Matterhorn Rehabilitation Hospital, Hiroshima 737-0046, Japan; matter@jasmine.ocn.ne.jp

**Keywords:** urinary incontinence, older adults, physical activity, mental health, health-related quality of life, well-being

## Abstract

Urinary incontinence (UI) is a major social problem for older adults and leads to a decline in health-related quality of life (HRQoL), mental health, and physical activity. This study assessed the prevalence and symptoms of UI among older adults discharged from the hospital in Japan and investigated the association of UI symptoms with physical activity, HRQoL, and subjective well-being (SWB). By an international consultation, the Incontinence Questionnaire Short Form (ICIQ-SF) that assesses UI severity, was developed. Self-administered questionnaires were used to assess physical activity, HRQoL, SWB, and social demographic characteristics of the participants. In total, 145 participants (valid response rate, 48%; mean age, 78.6 ± 7.6 years) were included in the analysis. Multivariate logistic regression analysis was performed to identify significant factors associated with the presence of UI. Significant decreases in physical activity, HRQoL, and SWB were observed in patients with UI compared with those without UI (*p* < 0.05). Multivariate analysis revealed that age, number of reported conditions, and decreased SWB were associated with UI (*p* < 0.05). UI was associated with less physical activity and decreased mental health status in older adults (especially decreased SWB). Health-promoting measures for older adults with UI are essential for maintaining their well-being and extending healthy life expectancy.

## 1. Introduction

The population of Japan is aging more rapidly than that of any other country, and there is an urgent need to address various health issues that are expected to arise in the aging society. One of the health issues that needs to be addressed is the increasing proportion of people with pelvic floor dysfunctions, such as urinary incontinence (UI) and pelvic organ prolapse [1,2]. According to an epidemiological survey of men and women aged over 40 years in Japan, 41.7% were estimated to have UI symptoms [3]. Despite the prevalence of UI, many patients are reluctant to discuss their urinary symptoms or incontinence. The symptoms of UI, such as stress or urge UI, are often caused by functional impairment of the pelvic floor muscles. Hence, previous studies have shown that pelvic floor muscle exercise interventions [4,5] and psychological counseling [6] are effective methods for relieving UI symptoms.

UI is a common condition that significantly reduces a patient’s quality of life (QoL) and is one of the factors placing a heavy burden on family caregivers [7,8,9]. Smith [10] reported an interrelationship between UI symptoms and health-related QoL (HRQoL) and subjective well-being (SWB) in women aged 45–60 years with UI; UI symptoms do not directly affect SWB, but the symptoms affect HRQoL, which in turn may lead to a decline in SWB.

To the best of our knowledge, the association of the presence of UI with physical activity, HRQoL, and SWB in older adults discharged from the hospital remains unclear. In addition, there is paucity of studies exploring adverse psychosocial effects of UI in Japan. Therefore, identifying factors that affect these parameters may suggest future public health measures that may help to maintain community QoL and SWB, and prevent exacerbations of decline in physical activity due to the narrowing of the range of activity. Thus, this study assessed the prevalence and symptoms of UI among older adults in Japan and investigated the effect of UI symptoms on physical activity, HRQoL, and SWB.

## 2. Materials and Methods

### 2.1. Study Design and Setting

This study used a cross-sectional design. The study has been reported according to the recommendations of the Strengthening the Reporting of Observational Studies in Epidemiology (STROBE) [11].

### 2.2. Participants and Survey Procedures

To target older adults discharged from hospitals, we randomly selected 400 patients from the patient database of the Matterhorn Rehabilitation Hospital in Kure city, Hiroshima Prefecture. The inclusion criteria were as follows: discharged to own home from the convalescent rehabilitation hospital between January 2017 and January 2020, visited our clinic at least once per week for >2 months during the same period, and aged > 60 years at the time of the survey. We excluded patients who could not respond to all items on a questionnaire, were discharged from the hospital to a nursing home or care facility, and could not walk independently. All patients provided informed consent to participate in the study. The study protocol met the requirements of the Declaration of Helsinki and was approved by the Ethical Committee for Epidemiology of Hiroshima University (approval number E-2179). The survey was administered as an anonymous, self-reported, postal questionnaire. The survey period was between 30 March 2020 and 30 May 2020.

### 2.3. Measurements

#### 2.3.1. Social Demographics

We recorded data on numerous participant characteristics, including age, sex, height, weight, body mass index, family structure (living alone, living as an older adult couple, or living with family), and medical history (hypertension, diabetes, cardiovascular, cerebrovascular, and orthopedic disease). Medical histories were obtained as physician-diagnosed medical conditions.

#### 2.3.2. Frequency and Severity of UI

An international consultation developed the Incontinence Questionnaire Short Form (ICIQ-SF) that assesses both the severity of UI and QoL of patients with UI; we used the validated Japanese version of the ICIQ-SF [12]. The ICIQ-SF comprises six questions that assess the frequency and severity of UI, and the degree of its interference with daily life. Patients with and without incontinence were classified into “UI-present” and “UI-absent” groups, respectively. Additionally, The ICIQ-SF includes a series of questions concerning the circumstances in which urine leakage occurs. We drew a distinction between stress UI (urine ‘leaks when you cough or sneeze’ or ‘leaks when you are physically active/exercising’), urge UI (urine ‘leaks before you can get to the toilet’) and mixed UI (presence of both) [13].

#### 2.3.3. Assessment of Higher-Level Functional Capacity

The Tokyo Metropolitan Institute of Gerontology Index of Competence measures higher functional capacity [14] and uses a multidimensional 13-item scale with three subscales, intellectual activities (four items), and social roles (four items) [15]. The responses to each question were set as “yes” (able to do) or “no” (unable), with a score of 1 for “yes” and 0 for “no.” Functional health was classified as independent (13 points) or dependent (≤ 12 points) [16].

#### 2.3.4. Level of Physical Activity

Participants’ levels of physical activity were determined using the self-administered and self-reported Physical Activity Questionnaire for Elderly Japanese [17]. This assessment was specifically designed to measure typical activity patterns of daily life. The score was calculated as the metabolic equivalent of task hours per week.

#### 2.3.5. HRQoL

Participants’ HRQoL was assessed using the Japanese version of the Medical Outcome Study 12-Item Short-Form Survey v2 (SF-12v2) questionnaire. SF-12v2 is a well-known globally relevant questionnaire with high reliability and validity for measuring HRQoL [18]. SF-12v2 comprises 12 questions that are related to eight different domains of QoL: physical functioning, role limitations due to physical illness, bodily pain, general health perceptions, vitality, social functioning, role limitations due to emotional problems, and mental health. The score in each domain was transformed to a numerical score, ranging from 0 to 100, and separately calculated using algorithms for the physical component summary score (PCS) and the mental component summary scores (MCS); higher scores reflected better self-perceived health.

#### 2.3.6. SWB

SWB was assessed using the Japanese version of the World Health Organization Five Well-being Index (WHO-5-J). WHO-5-J, a useful measure of the mental health, for older adults over a 2-week period, comprises the following five items: (1) felt cheerful and in good spirits, (2) felt calm and relaxed, (3) felt active and vigorous, (4) woke up feeling fresh and rested, and (5) daily life filled with things that interest me [19]. The answers to each item were evaluated using a six-point Likert scale, ranging from 0 to 5, with the maximum score being 25; higher scores indicated better SWB.

### 2.4. Statistical Analysis

Continuous data are presented as means ± standard deviations or medians (min, max). Before analysis, the Shapiro–Wilk test was conducted to check normality. Differences between the UI-absent and UI-present groups were detected using the Mann–Whitney *U*-test (continuous variables) or χ^2^ test (nominal variables).

To identify factors associated with the presence of UI, a multivariate analysis was performed using logistic regression. The sample size for the logistic regression analysis was previously estimated using six independent variables. A previous study suggested that the number of participants per independent variable should be ≥10 [20]; therefore, at least 60 participants were required in each of the study groups (UI-absent and UI-present) to allow a stratified analysis. Assuming a 50% valid response rate, the sample size for the questionnaire distribution was estimated to be 240 for both the UI-absent and UI-present groups. Before the multivariate analysis, a univariate analysis for each variable was conducted; variables showing significant differences (*p* < 0.10) in the univariate analysis were included as explanatory variables in the subsequent multivariate analysis. To minimize type II errors, the entry probability for logistic analysis was set at 0.10-level of significance rather than at 0.05 level because of the sample size and number of variables. The model was simplified in a backward stepwise (Wald) manner by removing variables with *p*-values > 0.05. Odds ratios (ORs) and 95% confidence intervals (CIs) were calculated for dependent variables associated with independent variables. The possibility of multicollinearity of the independent variables in the multivariate regression analysis was assessed by calculating the variance inflation factor. All data were analyzed using IBM SPSS Statistics for Mac, version 23.0 (IBM Corp., Armonk, NY, USA). A *p*-value < 0.05 was considered statistically significant, except for the entry probability in the logistic analysis mentioned above.

## 3. Results

### 3.1. Participant Selection

A total of 174 participants (43.4%) responded. At the time of analysis, 10 participants had already died and 10 had been transferred to nursing homes or care facilities and were excluded from the analysis. Furthermore, nine respondents provided incomplete or insufficient responses. Thus, 145 participants (valid response rate, 36.3%) were included in the analysis. Based on their UI questionnaire responses, the participants were assigned to the UI-absent (*n* = 73) or UI-present (*n* = 72) group.

### 3.2. Sociodemographic Data

The sociodemographic data of the participants according to the presence of urinary symptoms are presented in Table 1. The mean age of the participants was 78.6 ± 7.6 years, and 69.0% (*n* = 100) of participants were females. More than half (*n* = 75, 54.0%) of the included participants reported the presence of hypertension; somewhat less than half of the participants (*n* = 63, 43.4%) reported the presence of orthopedic disease. The majority of respondents (*n* = 105, 72.4%) lived alone or as members of older adult couples. A comparison of the two groups showed that age (*p* = 0.002, effect size = 0.26) and the number of patients with diabetes (*p* = 0.007, effect size = 0.23) were significantly higher in the UI-present group than in the UI-absent group.

### 3.3. UI Symptoms

Table 2 shows a summary of the responses to questions in the ICIQ-SF questionnaire. In the UI-present group, 28 (38.9%) participants reported having urine leakage approximately once a week, most frequently involving small volumes (*n* = 55; 76.4%), and frequently occurring before arriving in the bathroom (*n* = 48; 55.7%).

### 3.4. Functional Capacity, Physical Activity, HRQoL, and SWB

**Figure 1:**
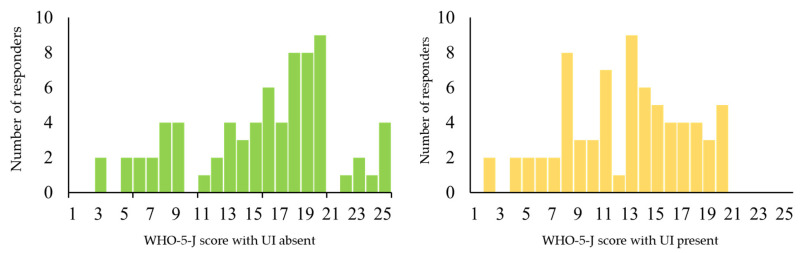
Distribution of the World Health Organization 5 Well-being Index (Japanese version, WHO-5-J) scores. Left: UI-absent group (*n* = 73), Right: UI-present group (*n* = 72).

There were significantly lower mean physical activity outcomes, HRQoL scores, and WHO-5-J scores in the UI-present group than in the UI-absent group (*p* < 0.05; Table 3). The distribution of the WHO-5-J scores is shown in Figure 1. The kurtosis and skewness were −0.463 and −0.497, respectively, in the UI-absent group and −0.230 and −0.686, respectively, in the UI-present group.

### 3.5. Association of UI with Geriatric Assessment Domains

From the univariate analysis results, age (OR, 1.069; 95% CI, 1.021–1.19; *p* < 0.01), number of medical conditions (OR, 1.652; 95% CI, 1.162–2.350; *p* < 0.01), physical activity (OR, 0.995; 95% CI, 0.989–1.001; *p* < 0.10), HRQoL PCS (OR, 0.97; 95% CI, 0.940–0.995.; *p* < 0.05), HRQoL MCS (OR, 0.950; 95% CI, 0.916–0.985; *p* < 0.01), and SWB scores (OR, 0.892; 95% CI, 0.834–0.954; *p* < 0.01) were associated with the presence of UI (data not shown in the table).

Table 4 summarizes the results of the multiple regression analysis of the association between the presence of UI and independent variables. Age, number of reported medical conditions, and SWB were associated with the presence of UI, but physical activity and HRQoL PCS and MCS scores were not were associated with the presence of UI.

## 4. Discussion

The main finding of this study indicated that UI was related with age, diabetes mellitus, less physical activity, decreased HRQoL, and decreased SWB in Japanese older adults discharged from the hospital, and that decreased mental health status was strongly associated with UI.

This study showed that the rate of UI in older adults was 49.7%, which is similar to that previously reported [21]. The prevalence of UI in women in our study was 72.2%, compared with a national prevalence of 55.5% that was observed in another study of a population with a mean age of 51.7 ± 9.5 years [22]. The mean age of the participants in our study was 78.6 ± 7.6 years, suggesting a higher prevalence of UI in the older age group. Thus, these studies appear to confirm that older members of the population are more likely to experience UI and the attendant decreases in QoL and SWB. This highlights the need for attention to this correlation, particularly in an aging society.

In Japan, many patients are reluctant to discuss UI, leading to unresolved physical, functional, and psychological morbidities and diminished QoL. Many researchers have indicated that the mental health of individuals with UI is related to UI severity [21,23,24]. Most patients with UI believe that incontinence is a natural process of aging and that there is no clear management approach or treatment for it [21]. Thus, individuals with UI are embarrassed to discuss their urinary incontinence symptoms with their family members or their health care providers. Hence, health care providers should encourage older adults to ask questions about UI. Education regarding UI, as a disease, should be increased to provide appropriate information and treatment options to older adults.

A comparison of parameters, other than sociodemographic and clinical parameters, between the UI-absent and UI-present groups showed poorer functional health, lower physical activity, and lower scores in both the physical and psychological aspects of the HRQoL tool in the UI-present group. These outcomes may lead to lower self-esteem; interfere with sexual activity; and restrict social contact, household chores, and work among older adults with UI [25]. These individuals have difficulty leaving the house because of a constant need to go to the bathroom, a need to know where the bathroom is when shopping or traveling, and they experience fear and frustration regarding being unable to get to the bathroom without an incontinence episode. This can greatly hinder their activities of daily living and leisure time [22,26]. QoL has been reported to decrease significantly as the severity of UI increases [27,28]. Our findings support the notion that older adults with UI have significantly reduced physical and psychological aspects of their HRQoL, compared with individuals without UI.

The present study indicated a negatively skewed and leptokurtic distribution of WHO-5-J scores in the UI-present group compared with that in the UI-absent group. This may mean that the peak of the distribution may have shifted from high to medium scores for the UI-present group, implying a lower SWB when compared with older adults without UI. These results also suggest that the presence of UI symptoms affects the mental health of older adults and reduces their sense of well-being. Odlum et al. [29] reported that women with UI had significantly higher scores for depression than men, which is consistent with a higher likelihood of depression and anxiety disorders. Therefore, continuous mental health care should be provided to older individuals with UI, especially older women.

In this study, the presence of UI was significantly associated with older age, the diabetes mellitus, and lower SWB among older adults in Japan. This emphasizes the importance of comprehensive geriatric assessments of other previous and current medical problems among older adults with UI symptoms. The association between diabetes and UI is consistent with that observed in a previous study [30]. Furthermore, the present results showed a significant association between UI and SWB. The results suggest that UI affects QoL and leads to a decrease in well-being during daily life. Regardless, an understanding of the association between UI and SWB in older adults is still in its infancy. However, in addition to SWB, age and the number of conditions reported in the patients’ medical histories contributed significantly to UI; thus, observing the interaction of these characteristics with different aspects of UI is possible. Overall, our results suggest that UI in older adults can limit their behaviors, affect their activities of daily living, and have psychosocial effects (e.g., negatively impacting their SWB).

The present study design allowed an examination of factors associated with the presence or absence of UI symptoms, including physical characteristics, physical activity, HRQoL, and SWB, in older adults. However, the study also had several limitations. First, the measures of physical activity, HRQoL, and SWB were self-reported and retrospective, which may have led to recall bias. Second, there were aspects of sexuality and psychology that were not completely considered in this quantitative study; our understanding of such issues could possibly be deepened by including a pre- and post-psychotherapy intervention comparison. Third, results from current study cannot perfectly apply to the community-dwelling people because participants in this study was community-dwelling people who had discharged from hospital. Fourth, the study sample was very limited compared with samples of other similar studies that have included hundreds of participants so that this study needed to narrow down the explanatory variables by Bayesian variable selection method.

## 5. Conclusions

According to our study, older adults with UI symptoms discharged from the hospital are significantly less physically active compared with those without UI symptoms, which significantly affects their HRQoL and SWB. This suggests that promotion of psychological and social supports (such as reducing the stigma of UI and supporting those with UI to improve their self-care skills) are essential for preventing physical inactivity owing to UI symptoms and improving the individual SWB. Furthermore, measures that promote mental and physical health in such individuals may prevent the exacerbation of decline in physical activity due to narrowing of the range of activity in the presence of UI and help to maintain community well-being. Maintaining physical activity and psychological health of older adults with UI symptoms will require providing: (1) knowledge that UI is a disease that can affect anyone with aging and treatment options; (2) appropriate literacy education that UI symptoms is not an embarrassing disease to achieve better HRQoL and SWB; (3) interventions for the functional improvement of pelvic floor muscles by exercise; and (4) social resources such as local counseling centers and health classes for older adults who are mentally depressed, especially when the depression is related to UI symptoms.

## Figures and Tables

**Table 1:** Sociodemographic data of the study participants according to the presence of urinary symptoms.

Participant Characteristics		Total	UI-Absent	UI-Present	*p*-Value
		*n* (%)	*n* (%)	*n* (%)	
Sex	Female	100 (69.0)	48 (65.8)	52 (72.2)	0.474
	Male	45 (31.0)	25 (34.2)	20 (27.8)	
Age (years)	60–64	9 (6.2)	6 (8.2)	3 (4.2)	
	65–69	9 (6.2)	3 (4.1)	6 (8.3)	
	70–74	23 (15.9)	17 (23.3)	6 (8.3)	
	75–79	36 (24.8)	21 (28.8)	15 (20.8)	
	80–84	33 (22.8)	16 (21.9)	17 (23.6)	
	85–89	25 (17.2)	8 (11.0)	17 (23.6)	
	90+	10 (6.9)	2 (2.7)	8 (11.1)	
	Mean	78.6 ± 7.6	76.8 ± 7.1	80.4 ± 7.8	0.002
Physician-diagnosed medical conditions	None	30 (20.7)	19 (26.0)	11 (15.3)	0.151
	Diabetes	19 (14.1)	4 (5.8)	15 (22.7)	0.007
	Hypertension	75 (54.0)	34 (49.3)	41 (58.6)	0.246
	Cardiovascular	19 (13.1)	8 (11.0)	11 (15.3)	0.471
	Cerebrovascular	20 (13.8)	8 (11.0)	12 (16.7)	0.346
	Orthopedic	63 (43.4)	27 (37.0)	36 (50.0)	0.133
Family structure	Alone	41 (28.3%)	20 (27.4%)	21 (29.2%)	0.204
	Older adult couple	64 (44.1%)	37 (50.7%)	27 (37.5%)	
	With family	40 (27.6%)	16 (21.9%)	24 (33.3%)	

Data are expressed as means ± SD, or *n* (%).

**Table 2:** Responses of patients with urinary symptoms to the ICIQ-SF questionnaire.

ICIQ-SF		UI-Present (*n* = 72)	Stress-UI (*n* = 32)	Urge-UI (*n* =19)	Mixed-UI (*n* = 7)	Other- UI (*n* = 7)
Interferes with quality of life score (points)		2.51 ± 2.13	2.63 ± 2.38	1.68 ± 1.20	3.07 ± 2.09	3.14 ± 2.67
Total score (points)		7.28 ± 3.64	7.56 ± 3.66	5.21 ± 1.72	8.43 ± 3.57	9.29 ± 5.38
Urine loss frequency	Once per week	28 (38.9%)	11 (34.4%)	14 (73.7%)	1 (7.1%)	2 (28.6%)
	Several times per week	16 (22.2%)	7 (21.9%)	3 (15.8%)	5 (35.7%)	1 (14.3%)
	Once per day	11 (15.3%)	6 (18.8%)	1 (5.3%)	2 (14.3%)	2 (28.6%)
	Several times per day	15 (20.8%)	7 (21.9%)	1 (5.3%)	6 (42.9%)	1 (14.3%)
	All the time	2 (2.8%)	1 (3.1%)	0 (0.0%)	0 (0.0%)	1 (14.3%)
Volume of urine lost	Small	55 (76.4%)	23 (71.9%)	18 (94.7%)	11 (78.6%)	3 (42.9%)
	Moderate	16 (22.2%)	9 (28.1%)	1 (5.3%)	3 (21.4%)	3 (42.9%)
	Large	1 (1.4%)	0 (0.0%)	0 (0.0%)	0 (0.0%)	1 (14.3%)
Urine loss *	Before getting to the bathroom	48 (55.7%)	32 (100%)	0 (0.0%)	14 (100%)	0 (0.0%)
	When coughing or sneezing	35 (39.2%)	0 (6.3%)	19 (61.3%)	12 (85.7%)	0 (0.0%)
	Sleeping	8 (8.9%)	2 (38.9%)	0 (0.0%)	2 (14.3%)	4 (57.1%)
	While performing physical activities	6 (6.3%)	0 (0.0%)	0 (0.0%)	5 (35.7%)	0 (0.0%)
	When finishing urinating and getting dressed	4 (3.8%)	1 (3.1%)	1 (5.3%)	2 (14.3%)	0 (0.0%)
	No obvious reason	6 (6.3%)	2 (38.9%)	0 (0.0%)	2 (14.3%)	2 (28.6%)
	All the time	0 (0.0%)	0 (0.0%)	0 (0.0%)	0 (0.0%)	0 (0.0%)

ICIQ-SF, International consultation developed Incontinence Questionnaire Short Form. Data are expressed as means ± SD or *n* (%). * means multiple choices allowed.

**Table 3:** Functional capacity, physical activity, HRQoL, and SWB according to the presence of urinary symptoms.

Variables	Total (*n* = 145)	UI-Absent (*n* = 73)	UI-Present (*n* = 72)	*p*-Value	Effect Size (Z)
Functional capacity	8.7 ± 4.1	9.4 ± 3.7	7.9 ± 4.3	0.066	0.15
Physical activity					
Total	54.3 ± 54.7	61.8 ± 57.2	46.7 ± 51.3	0.043	0.17
Transportation	6.7 ± 8.6	7.3 ± 8.4	6.0 ± 8.7	0.145	0.12
Exercise or sports	12.4 ± 19.8	14.6 ± 23.2	10.1 ± 15.4	0.046	0.18
Housework	27.1 ± 35.4	30.0 ± 37.8	24.2 ± 32.8	0.224	0.14
Labor	8.2 ± 20.0	9.9 ± 22.3	6.4 ± 17.4	0.381	0.07
HRQoL					
PCS (0–100)	37.0 ± 12.0	39.3 ± 12.1	34.7 ± 11.6	0.026	0.19
MCS (0–100)	51.6 ± 9.7	53.9 ± 9.6	49.3 ± 9.2	0.006	0.23
SWB	14.0 ± 5.4	15.5 ± 5.6	12.4 ± 4.7	<0.001	0.30

UI, Urinary incontinence; HRQoL, health-related quality of life, SF-12v2 score; PCS, physical component summary; MCS, mental component summary; SWB, subjective well-being, WHO-5-J score. Data are expressed as means ± SD.

**Table 4:** Association of UI with geriatric assessment domains.

Variables	β	SE	Wald	df	*p*-Value	Odds Ratio	95% CI	
							Lower	Upper
Age	0.063	0.025	6.440	1	0.011	1.065	1.015	1.119
Number of medical conditions	0.468	0.190	6.062	1	0.014	1.566	1.100	2.316
Physical activity	0.001	0.004	0.020	1	0.889	1.001	0.993	1.008
HRQoL PCS	−0.006	0.019	0.118	1	0.732	0.994	0.958	1.031
HRQoL MCS	−0.029	0.023	1.572	1	0.210	0.972	0.929	1.016
SWB	−0.111	0.036	9.337	1	0.002	0.895	0.835	0.961

UI, urinary incontinence; SE, standard error; CI, confidence interval; DM, diabetes mellitus; HRQoL, health-related quality of life, score of SF-12v2; PCS, physical component summary; MCS, mental component summary; SWB, subjective well-being, score of WHP-5-J score. β, partial regression coefficient. SE, standard error. df, degree of freedom. Variance inflation factor: age, 1.24; DM, 1.05; Physical activity, 1.42; PCS, 1.39; MCS, 1.64; SWB, 1.80.
